# Speed in Operations

**Published:** 1900-08-25

**Authors:** 


### SPEED IN OPERATIONS.

One of the lessons learned by some of those who went to the Paris Congress was that it is possible to do operations in a much shorter time than they usually occupy at the present day. The modern surgeon has taken advantage to the full of the ease, the comfort, and the security offered by the fact that his patient cannot feel. He takes his time, and the question must now and then arise in the mind of the observer whether he is not a little bit more leisurely in his movements than is quite desirable. That there is another way of doing things was made manifest enough to those who attended the clinique of Dr. Doyen, and saw the rapidity with which he got through his work. Of course, there is a good side and there is a bad side to all this. It is being every day more fully recognised that prolonged anæsthesia is a serious addition to the dangers of any operation, and there is a certain truth in Doyen's saying that to the surgeon "time is life." Most surgeons will admit that so far as it is possible to shorten operative procedures by the use of mechanical appliances, as by electrical drills and saws, in place of the old hand-driven instruments, and by the attainment of a superlative skill in technique by prolonged and untiring practice, all the increased speed so gained is purely to the advantage of the patient. But it is open to doubt whether the mental processes by means of which the surgeon is enabled to come to a decision on points of difficulty which may arise in the course of an operation can be equally hastened, and, in view of the fact that the exact procedure required cannot always be completely formulated until after an operation has well advanced, we may question whether such speeding of operative work as that with which we are now threatened under the stimulating influence of the kinematograph, by which every moment given to thought or lost in hesitation is accurately recorded against one, is entirely beneficial. In those few operations in regard to which the exact steps to be followed can be mapped out and laid down beforehand a very high speed can be attained by careful practice, but unless a man's mind is by nature as agile as his fingers can be made by practice, any attempt to operate at lightning speed will certainly lead only to disaster.

